# Clinical outcomes of subtalar arthroereisis for the treatment of stage 1 flexible progressive collapsing foot deformity

**DOI:** 10.1007/s00590-024-04007-4

**Published:** 2024-05-30

**Authors:** Thomas L. Lewis, Thomas A. J. Goff, Robbie Ray, Jagwinder Dhaliwal, David Carmody, Andrew P. Wines

**Affiliations:** 1 King’s Foot and Ankle Unit, King’s College Hospital NHS Foundation Trust, London, UK; 2 Mid Yorkshire Hospitals NHS Trust, Wakefield, UK; 3 Sandwell and West Birmingham Hospitals NHS Trust, Birmingham, UK; 4 North Sydney Orthopaedic and Sports Medicine Centre, Wollstonecraft, Australia

**Keywords:** Subtalar arthroereisis, Flatfoot, Adult flatfoot, Pes planus, Tibialis posterior, Hind foot reconstruction, Subtalar joint, Arthrodesis, Screw removal, Progressive collapsing foot deformity

## Abstract

**Background:**

The use of subtalar arthroereisis as an adjunct to the surgical treatment of stage 1 flexible progressive collapsing foot deformity (PCFD) is controversial. The aim was to investigate the clinical outcomes and report the implant removal rate of subtalar arthroereisis as an adjunct for stage 1 PCFD.

**Methods:**

A retrospective study of 212 consecutive feet undergoing operative management of stage 1 PCFD with adjunctive subtalar arthroereisis between October 2010 and April 2018. The primary outcome was the Foot and Ankle Outcome Score (FAOS). Secondary outcomes included Foot and Ankle Disability Index (FADI), Euroqol-5D-5L Index and implant removal rate.

**Results:**

Post-operative clinical FAOS outcomes were collected for 153 feet (72.2%). At mean 2.5-year follow-up, the mean ± standard deviation FAOS for each domain was as follows; Pain: 81.5 ± 18.5, Symptoms: 79.5 ± 12.9, Activities of Daily Living: 82.5 ± 15.4 and Quality of Life: 64.2 ± 23.7. EQ-5D-5L Index was 0.884 ± 0.152. Pre-operative scores were available for 20 of these feet demonstrating a statistically significant improvement in all FAOS, FADI and EQ-5D-5L domains (*p* < 0.05). The implant removal rate for persistent sinus tarsi pain was 48.1% (*n* = 102).

**Conclusion:**

Use of a subtalar arthroereisis implant as an adjunct to conventional procedures in stage 1 flexible PCFD can result in significant improvement in pain and function. Patients should be counselled as to the relatively frequent rate of subsequent implant removal.

**Level of evidence:**

IV.

## Introduction

Progressive collapsing foot deformity (PCFD) affects 3% of adults and is characterised by hind foot valgus, collapse of the medial longitudinal arch and varying degrees of forefoot abduction and supination [1]. It often results from tibialis posterior tendon dysfunction and can lead to debilitating pain, difficulty mobilising and gait abnormality [1].

Subtalar arthroereisis has an established use in paediatric flexible flatfoot deformity with good clinical and radiographic results [2–4]. The function of the implant is to limit excessive subtalar joint pronation, decreasing the tendency for the talus to rotate planto-medially, reducing strain on the medial structures. There are three main types of implant: axis-altering, which limits internal rotation of the calcaneus and modifies the subtalar joint axis; impact-blocking, which limits anterior glide and internal rotation of the talus and self-locking, which limits talar adduction and plantarflexion [5]. In adults, subtalar arthroereisis can be used as an adjunct to well-established operative techniques used in the correction of a flatfoot deformity [1, 5]. Restriction of excessive eversion, particularly during early post-operative rehabilitation and initial weight-bearing, may be advantageous to the healing/reinforcement of operated medial structures [6] and, thus, overall clinical outcomes [7, 8].

A recent systematic review of nine studies assessed the outcomes of 190 feet and found that following treatment with subtalar arthroereisis, either as an adjunct or alone, both the clinical and radiological outcomes improved significantly [1]. There are, however, methodological issues with existing studies including underpowered studies with small sample numbers, single-surgeon series and variable reporting of clinical outcomes. There are also concerns regarding the need for implant removal necessitating further surgery.

The aim of this study was to present the clinical outcomes of subtalar arthroereisis as an adjunct for the surgical management of stage 1 flexible PCFD and report the implant removal rate.

## Methods

### Study design

This was a retrospective observational study of all consecutive patients undergoing operative management of stage 1 flexible PCFD with an adjunctive subtalar arthroereisis implant inserted with a minimum of 6 months follow-up. Stage 1 flexible PCFD was defined as *“a correctable (flexible) flat foot deformity with varying degrees of heel valgus, midfoot abduction and forefoot supination” *[9–12]. This study has been reported following the STROBE template [13] for observational studies.

### Setting

This study was conducted in a single centre in Australia with all operations carried out by either DC or AW. Due to the nature of the study, blinding was not possible in this study.

### Participants

All consecutive patients diagnosed with stage 1 flexible PCFD who had failed a minimum of 6 months of non-operative management were offered surgical reconstruction. Patients younger than 18 years of age, congenital foot deformities, radiographic evidence of subtalar or transverse tarsal joint arthritis, rigid (non-correctable) flatfoot, patients with incomplete medical records and those with less than 6 months of post-operative follow-up were excluded.

### Outcome measures

The primary outcome was the Foot and Ankle Outcome Score (FAOS) [14]. Four components of this score (Pain, Symptoms, Quality of Life and Activities of Daily Living) were collected. The FAOS has previously been validated for the assessment of Adult Acquired Flatfoot Deformity (AAFD) [15]. Secondary outcomes included the Euroqol-5D-5L health-related quality of life score, the Foot and Ankle Disability Index (FADI) and implant removal rate.

### Data sources

Data including patient age, sex, procedure laterality, implant removal and post-operative Patient Reported Outcome Measures (PROMs) were obtained from medical records for all patients operated on from 2010 to 2018. In 2017, pre-operative clinical outcome data started to be routinely collected during outpatient clinic appointments and were available for analysis. In 2019, all patients without post-operative PROMs or implant removal data were contacted via telephone, post and/or email for follow-up. Radiographic measures were not consistently documented or available for retrospective review (due to the nature of the healthcare system) during the study period so have not been reported here.

### Operative technique

**Fig. 1:**
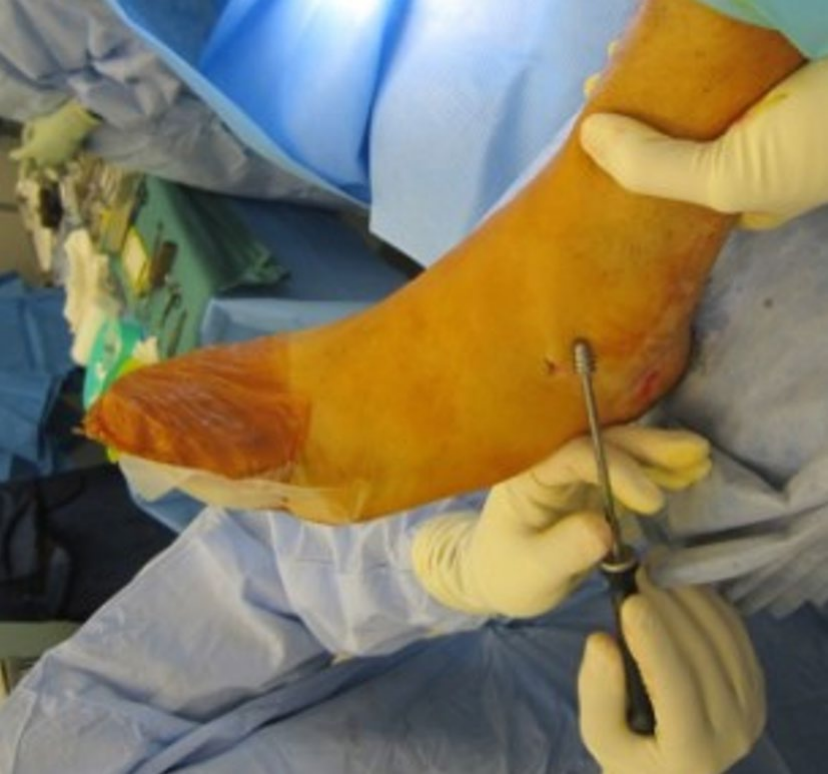
Intra-operative clinical photograph demonstrating implant insertion

**Fig. 2:**
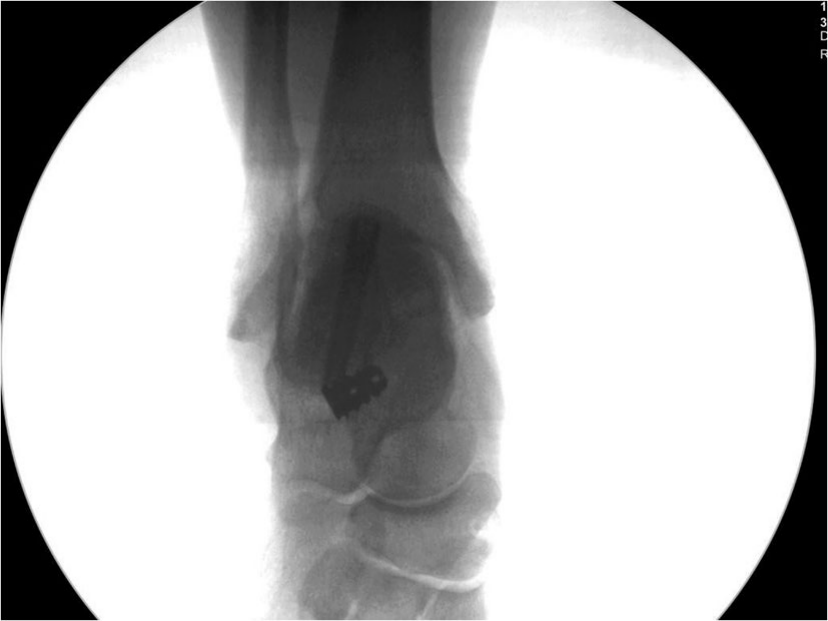
Dorsal–plantar intra-operative radiograph demonstrating sinus tarsi implant position

**Fig. 3:**
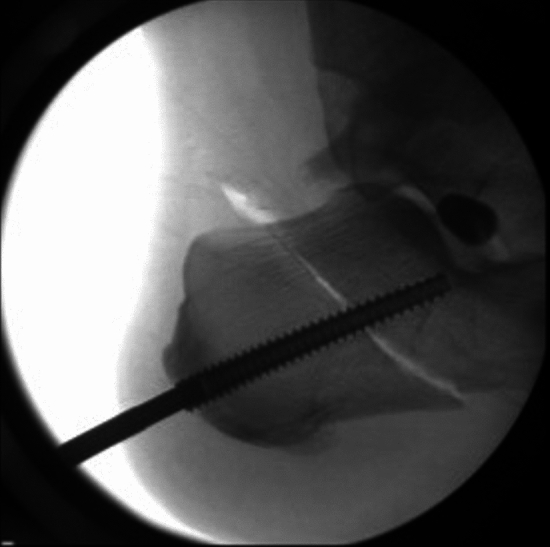
Lateral intra-operative radiograph demonstrating sinus tarsi implant position

**Fig. 4:**
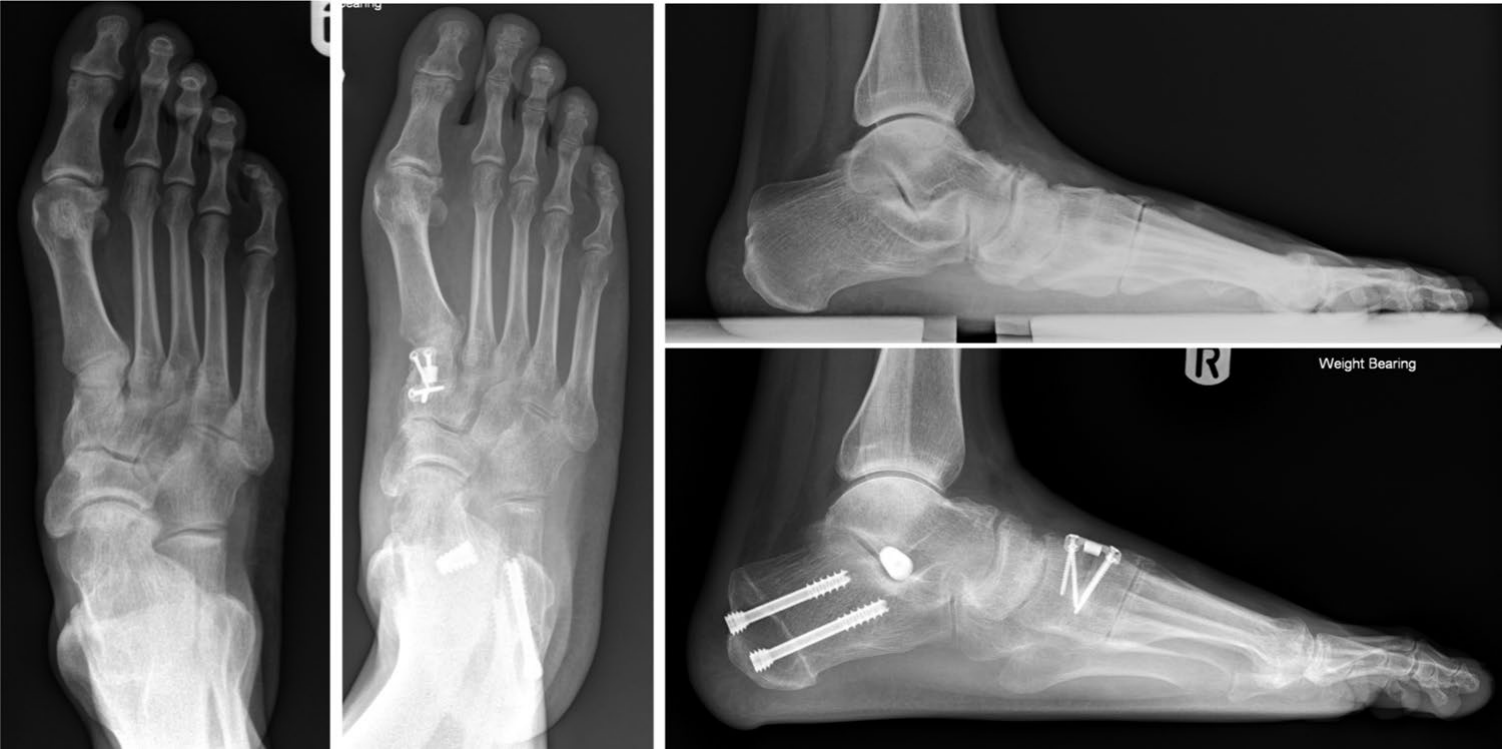
Pre- and post-operative weight-bearing radiographs demonstrating the subtalar arthroereisis implant position just under the lateral shoulder of talus and approximately 50% of the way across the underside of talus

Surgery proceeded correcting all parts of the flatfoot deformity in sequence: ankle, hind foot, midfoot and forefoot. Either a percutaneous Achilles lengthening using the triple-cut technique or a medial approach gastrocnemius muscle recession was performed if needed to achieve 5° of ankle dorsiflexion with the hind foot neutral. A medialising minimally invasive or open calcaneal osteotomy using a low-speed high-torque burr corrected to neutral hind foot alignment and fixed with cannulated 7-mm compression screw fixation. The posterior tibial tendon was evaluated through a medial hind foot incision and augmented with transfer of the flexor digitorum longus tendon to the navicular. The subtalar arthroereisis implant^1^ was inserted via a 2-cm lateral incision over the sinus tarsi. A blunt-ended guide wire was passed from lateral to medial across the sinus tarsi. Cannulated trial sizes in diameters of 7–13 mm were used sequentially to determine correct implant size, assessing subtalar range of motion, correction of talar head flexion and abduction deformity and proper seating. Trial implant size was sequentially increased until opening of the subtalar joint on the lateral radiograph was visualised (indicating an oversized implant as per the operation technique). The final implant (one size smaller than the oversized trial) was implanted as shown in Figs. 1, 2, 3 and 4. If after screw insertion, residual forefoot supination persists, then a cotton osteotomy was performed. Additional first ray procedures for hallux valgus were excluded as these are frequently observed in PCFD and may have had a confounding effect on the outcomes of interest. Unfortunately, specific data regarding additional procedures were unavailable. Post-operative rehabilitation included initial 2 weeks non-weight-bearing mobilisation in a backslab, followed by progressive protected weight-bearing and physiotherapy in an aircast walking boot.

### Statistical methods

We performed the Shapiro–Wilk test and the Kolmogorov–Smirnov tests to assess for normality and found both tests indicated a normal distribution. As such, we used parametric tests for data analysis. Continuous data were analysed with paired and independent *t*-tests and reported using descriptive statistics. All statistical analyses were performed using the Python SciPy package [16]. Statistical significance was defined as a *P* value of less than 0.05.

## Results

### Participants

**Fig. 5:**
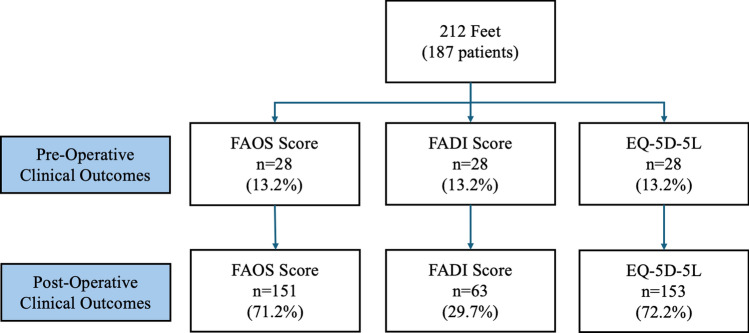
A flowchart of patient participation in this study including the proportion of pre- and post-operative clinical outcome data

Between October 2010 and April 2018, 187 consecutive patients (69 males and 118 females) underwent surgical reconstruction for stage 1 flexible PCFD. This comprised a total of 212 feet (126 left and 86 right). The mean age was 59.7 years (range 18.8–84.0, standard deviation (SD) ± 13.1 years). Thirteen patients (26 feet) underwent bilateral surgery on the same day. Figure 5 demonstrates the patient participant flowchart for the clinical outcome analysis. Of the 212 feet, 20 feet had matched pre- and post-operative scores, and 133 feet had post-operative scores only. The mean follow-up was 2.5 years (range 0.6–5.2, SD ± 1.3). A total of 133 feet also underwent Achilles tendon lengthening as part of the procedure to correct posterior chain tightness. The mean body mass index was 28.1 ± 4.3. The most common implant size was 9 mm (90.6% of cases).

### Clinical outcomes

Results were analysed based on two cohorts; patients with pre- and post-operative scores available (*n* = 21) and patients with only post-operative scores available (*n* = 143). Table 1 demonstrates the statistically significant improvement in clinical outcomes following surgical reconstruction of stage 1 flexible PCFD in patients with both pre- and post-operative scores (Fig. 5). The improvement for the FAOS domains exceeds the minimal clinically important difference (MCID) (Pain: 9.5, Symptoms: 0.3, ADL: 11.7 and QOL: 5.0) [17].

**Table 1:** Clinical outcomes following surgical reconstruction of stage 1 flexible progressive collapsing foot deformity

	Pre-operative (Cohort 1*)	Post-operative (Cohort 1*)	P value (between pre-/post-operative scores)	Post-operative (Cohort 2*)	P value (between post-operative cohorts)
	(Mean ± Standard Deviation)				
Number of feet	20	–	133	–	
Length of follow-up (years)	1.4 ± 0.4	–	2.3 ± 1.1	< 0.05	
Age (years)	60.7 ± 11.0	–	58.1 ± 13.5	0.49	
Gender (M:F)	7:13	–	45:74	–	
FAOS					
Pain	35.9 ± 17.2	73.0 ± 16.6	< 0.001	81.5 ± 18.5	0.05
Symptoms	71.8 ± 6.1	81.9 ± 9.6	< 0.001	79.5 ± 12.9	0.42
ADL	65.6 ± 14.6	84.0 ± 11.0	< 0.001	82.5 ± 15.4	0.66
QOL	26.6 ± 14.0	47.8 ± 20.1	0.001	64.2 ± 23.7	< 0.05
FADI					
Pain	52.0 ± 18.6	22.4 ± 16.9	< 0.001	17.8 ± 19.0	0.05
Activity	49.8 ± 22.0	23.8 ± 18.9	< 0.001	23.8 ± 20.6	0.94
EQ-5D-5L					
Index	0.693 ± 0.219	0.818 ± 0.136	< 0.05	0.884 ± 0.152	0.06

ADL, Activities of Daily Living and QOL, Quality of Life

^*^Cohort 1: patients with matched pre- and post-operative scores

^*^Cohort 2: patients with post-operative scores only

Table 1 also demonstrates the post-operative clinical outcomes of the two cohorts. Scores across the clinical outcomes in the post-operative outcome group only were comparable or statistically significantly improved compared to the matched cohort. Table 2 demonstrates the change in proportion of responses to the EQ-5D-5L by dimension. The EQ-5D-5L dimension data show that the main pre-operative issue is pain and issues with mobility.

**Table 2:** EQ-5D-5L dimension proportions of responses before and after stage 1 flexible progressive collapsing foot deformity with an adjunct arthroereisis implant

EQ-5D-5L dimension	Dimension level	Pre-operative	Post-operative	% change
Mobility	1	25.0	35.0	10
	2	5.0	40.0	35
	3	55.0	25.0	− 30
	4	15.0	0.0	− 15
	5	0.0	0.0	0
Self-care	1	90.0	90.0	0
	2	10.0	5.0	− 5
	3	0.0	0.0	0
	4	0.0	0.0	0
	5	0.0	5.0	5
Activities of daily living	1	25.0	35.0	10
	2	35.0	50.0	15
	3	35.0	15.0	− 20
	4	0.0	0.0	0
	5	5.0	0.0	− 5
Pain	1	5.0	10.0	5
	2	10.0	55.0	45
	3	60.0	35.0	− 25
	4	10.0	0.0	− 10
	5	15.0	0.0	− 15
Anxiety/depression	1	55.0	70.0	15
	2	15.0	20.0	5
	3	30.0	10.0	− 20
	4	0.0	0.0	0
	5	0.0	0.0	0

### Implant removal

Implant removal was considered for all patients complaining of ongoing sinus tarsi or lateral hind foot pain after 6 months from the index implantation procedure. A total of 102 implants (48.1%) were subsequently removed (90 patients, 30 males and 60 females), and the mean time for removal was 0.64 ± 0.41 years post-index implantation procedure. No implants were removed for infection or dislocation. Comparative analysis between cases that underwent implant removal and those that did not, demonstrated that there was a statistically significant difference in age (56.7 years (implant removed) vs 62.5 years (implant not removed), *p* < 0.05). We do not think that this is clinically meaningful but cannot confidently explain this difference. We hypothesise that a younger population may have a more mobile hind foot and experience symptomatic impingement leading to persisting hind foot pain and thus implant removal. There was no statistically significant difference in pre- or post-operative FAOS, FADI—Activity, FADI—Pain score or EQ-5D-5L Index score. Despite implant removal, four patients had persisting hind foot pain originating from the subtalar articulation with evidence of progressive arthrosis leading to subtalar arthrodesis surgery in all four cases at an average of 33 months from the index procedure. We do not believe that these were directly related to the arthroereisis screws as all four cases had radiographic signs of early osteoarthritis before primary reconstruction and implantation of the arthroereisis screw. One patient had ankle arthrodesis 24 months post-index procedure.

### Patient satisfaction

Patient satisfaction data were available for 88 feet (41.6%). About 52.3% (*n* = 46) of patients were “Very Satisfied”, 43.2% (*n* = 38) were “Satisfied”, 3.4% (*n* = 3) were “Neutral” and 1.1% (*n* = 1) were “dissatisfied”.

## Discussion

### Key results

This study reports on the largest series of subtalar arthroereisis for stage 1 flexible PCFD with validated clinical outcome measures and mean 2.5-year follow-up. In many cases, clinical outcomes were collected after implant removal allowing comparison between patients who underwent implant removal and those who did not. This showed that for the vast majority of clinical outcomes and health-related quality of life, there was no difference at final follow-up between patients who had the implant removed compared to those where it was retained. This is an important finding as this study reassures surgeons who can counsel patients who experience pain following subtalar arthroereisis that implant removal is unlikely to lead to worse clinical outcomes.

### Does adding an arthroereisis screw change clinical outcomes?

One particularly important question to consider is whether the addition of the subtalar arthroereisis screw changes the clinical outcomes. We were unable to directly assess this in this study as we did not have a comparator cohort; however, comparing to the previous studies of stage 1 flexible PCFD allows us to make comparisons to historical cohorts.

Coster et al. found an improvement in EQ-5D Index score from 0.55 to 0.76 two years following surgery for adult-acquired flatfoot deformity (without an arthroereisis screw) which is a greater improvement than we found in our study; however, our initial and final EQ-5D Index scores were both higher than their study [18]. Conti et al. reported FAOS 2 years following surgery for stage II AAFD for 143 consecutive feet and found similar scores to this study in the Symptoms and QOL domains [19]. Conti et al. reported generally higher post-operative scores compared to this study in the Pain and ADL domains. Soukup reported comparable FAOS following surgery with this study in the Pain, Symptoms and ADL domains whilst reporting lower QOL domain scores [20]. There does not appear to be convincing evidence from this study that use of an arthroereisis screw leads to improved or worse clinical outcomes in the surgical management of stage II AAFD compared to other studies. Further work should focus on randomised clinical studies comparing these two groups.

### FAOS score

There was a statistically significant improvement in the four domains of the FAOS that was assessed. The improvement in FAOS exceeded the minimal clinically important difference for each component suggesting that the improvement identified was clinically relevant [17]. Yasui et al. [21] also found a statistically significant improvement in FAOS domains with comparable post-operative scores to this study. There was no difference in FAOS in patients who had the implant removed compared to those where it was retained.

### Other outcome scores

To our knowledge, there are no other studies reporting the FADI or EQ-5D-5L scores for patients undergoing subtalar arthroereisis in the correction of PCFD. The change in EQ-5D-5L dimension proportions and EQ-5D-5L Index suggests that this procedure is associated with a corresponding improvement in overall health-related quality of life. There was no statistically significant difference in EQ-5D-5L Index score between patients who underwent implant removal and those where it was retained.

### Implant removal

Subtalar arthroereisis implant removal rates in the literature vary from 6.7–= to 58.3% as shown in Table 3 [1]. The incidence of implant removal in this series is higher than seen in many other studies. We believe that the removal rate in our study is more likely to represent the true implant removal rate as the small sample numbers in other (potentially underpowered) studies put them at risk of Type 2 error.

**Table 3:** Incidence of subtalar arthroereisis implant removal after treatment for stage II AAFD

Study	Country	Feet (*n*)	Mean age (*y*)	Mean follow-up (*m*)	Implants removed (%)	Implant
Present study	Australia	212	59.7 (18.8–84.0)	29	102 (48.1)	R2, R4, Wright medical CSI sinus tarsi spacer
Viladot et al. [22]	Spain	19	55 (20–76)	27	2 (10.5)	Kalix
Zaret et al. [23]	US	12	49 (19–82)	> 12	2 (16.7)	MBA
Needleman [24]	US	28	51 (28–74)	44	11 (39.3)	MBA
Cook et al. [25]	US	66*	30 (8–62)	> 12	22 (33.3)	NR
Garras et al. [26]	US	23*	23 (10–27)	53	3 (13.0)	NR
Baker et al. [27]	US	66	50 (20–80)	> 12	13 (19.7)	NR
Saxena et al. [28]	US	100	53 (19–80)	78	23 (23.0)	Prostop, Prostop-Plus, MBA, Kalix
Brancheau et al. [29]	US	60**	14 (5–46)	36	9 (15.0)	MBA
Viladot et al. [30]	Spain	35	55 (40–80)	48	13 (37.1)	NR
Graham et al. [31]	US	117	58 (22–85)	51	7 (6.0)	HyProCure
Ozan et al. [32]	Turkey	26	24 (18–35)	15	3 (11.5)	Horizon Biopro
Walley et al. [33]	US	15	51 (66–94)	40	1 (6.7)	Prostop
Zhu et al. [34]	China	24	48.8 (23–74)	30	14 (58.3)	Kalix

The predominant concern regarding the use of subtalar arthroereisis implants is the relatively high removal rate, which relates almost exclusively to persistent sinus tarsi pain and the need for implant removal. The aetiology is believed to be mechanical irritation [24], although it remains unclear why some patients develop this pain and others do not. Saxena et al. [28] evaluated factors linked to implant removal, finding a 22.1% incidence of removal, more frequently associated with implants sized > 11 mm, but with no relationship to patient age or performing Achilles tendon lengthening. This is in contrast with our study which found that age was a factor affecting implant removal rate and also the majority of the implants were smaller than 11 mm. There is evidence to suggest that the implant is no longer functional/necessary after sufficient time has lapsed for medial soft tissue healing, allowing implant removal without risking the surgical correction achieved [1, 7, 8]. Removal of the implant has been shown to help improve sinus tarsi pain whilst maintaining good functional clinical outcomes [1, 24] which this study indirectly corroborates. It is important to note that the reoperation rate of 48% for screw removal is a substantial limitation of the approach given removal involves potential morbidity, inconvenience, expense and anaesthesia associated with a further operation, and this should be taken into account when deciding whether or not to implant arthroereisis screws during a PCFD reconstruction. Further studies should be comparative in nature using validated outcome scores.

### Limitations

There are a number of limitations. The most important is the limited date regarding pre-operative clinical outcomes and short surgical follow-up time. Other limitations include the heterogeneous nature of the operative technique and lack of radiographic outcomes which would allow quantification of the deformity correction achieved (as well as indicate if there was any loss of correction following removal of the arthroereisis implant). Variability and the non-standardised time point when post-operative clinical outcome data were recorded may also confound results.

## Conclusion

Use of a subtalar arthroereisis implant as an adjunct to conventional procedures in flexible PCFD can result in significant improvement in pain and function. Patients should be counselled as to the frequent rate of subsequent implant removal. Implant removal does not change functional outcome compared to cases where the asymptomatic implant was retained.

## Footnotes

1 The subtalar arthroereisis implants used were of similar conical design—Wright Medical CSI, Signature Orthopaedics R2 and Double medical R4.
